# Abstracts from Foreign Exchanges

**Published:** 1886-01

**Authors:** 


### ABSTRACTS FROM FOREIGN EXCHANGES.

BY B. E. H.

The latest antisepticum is acetic acid in 1,0 to 0,25 per cent. It is recommended by Schulz of Greifswald.

---

Knowsley Thornton (*Medical Times and Gazette*) removed calculus from the kidney by a combination of abdominal and lumbar sections. By the abdominal incision the hand was enabled to press the kidney against the operating instruments from the back.

---

A very interesting discussion took place in the Berlin Medical Society following a paper of Mr. Posner on physiological albuminuria. It was conceded by all participants in the debate that the urine of healthy persons contains albumen, of course, in minimal quantities, which can be traced only by very exact and new methods. But even in larger percentage, so that the common tests are sufficient, albumen is present under certain circumstances; for instance, after steam baths, etc. It is very important to know that albumen in the urine does not, under all circumstances, prove disease of the kidneys. In addition, we report the statement of Vibert and Ogier (*Medical Record, December* 5, 1885,) that the urine drawn from the bladder of a cadaver is almost invariably albuminous, even when there was no lesion discoverable in any part of the uro-genital apparatus.

---

Hegar has removed ovaries and tubes for tuberculosis of these organs four times, making a correct diagnosis in advance. He gives the following as the leading points in diagnosis: Small, *hard*, irregular and angular nodules on the uterine end of the tubes, with soft and fluctuating swellings in gonorrhœa. In pyosalpinx, on the other hand, there are often signs of a fresh inflammation in the neighborhood, jelly-like masses, into which the examining finger easily makes impressions. Further on gonnococci may be found in the uterine or vaginal secretions. For tuburculosis the general condition may be taken into consideration, like hectic fever, nightsweats, etc. Further symptoms for tuberculous disease of the uterine appendages are bloating of abdomen, exudations in the lumbar regions. If the tubes are diseased, one can feel a string of nodules of different size, reaching from the womb to the pelvic wall; these nodules are little movable and adherent to the pelvis, the broad ligament or uterus. In operating for such cases, the surgeon has to be very careful not to burst the diseased organs.—*Centralblatt fuer Chirurgie.*

---

Woelfler (*Weimer Medical Wochenschirft*) recommends in supravaginal uterine amputation (for removal of fibroids, etc.) to sew the stump into the abdominal wound, and to treat it externally.

---

Talini Bassiano (*Parma*) operated for a echinococcus-cyst on the labium major of a woman.

---

Newmann (*Wiemer Medical Blaetter*) reports seven cases of sclerotic chancre excised; in three cases even the inguinal glands were removed, once already sixteen days after infection, without any success. This makes him believe that the favorable reports are given by unitarians, and that soft chancre has been excised.

---

Opitz (*Centralblatt fuer Gynackologie*) removed a piece of the infantile skull, which, after cranioclasty, had remained in the womb for two years, without causing much trouble.

---

At Berlin the new laboratory of Prof. R. Koch was opened on the third of November. Nearly all the places were taken on the first day.

---

In Wilhelmshaven, Germany, a wholesale poisoning by a kind of muscles occurred (*Mytilus edulis*), with some fatal cases. Koch could, to our surprise, detect no germs which could have been indicted. It was proven that the poison contained in the living animal is extremely fatal; it is probably a volatile alkaloid, and paralyzes the motor-centres very much like curara.

Prof. Von Bergmanns, of Berlin, statistics on extirpation of kidneys for carcinoma and sarcoma show forty-nine cases, of which nineteen were successful, twenty fatal. Thirty-seven cases were operated by laparotomy, with twenty-four deaths; eleven by lumbar incision, with five deaths.

